# Simultaneous femoral head reduction osteotomy (FHRO) combined with periacetabular osteotomy (PAO) for the treatment of severe femoral head asphericity in Perthes disease

**DOI:** 10.1186/s13018-022-03351-7

**Published:** 2022-10-20

**Authors:** Kaveh Gharanizadeh, Hadi Ravanbod, Amir Aminian, Seyed Peyman Mirghaderi

**Affiliations:** 1 Bone and Joint Reconstruction Research Center, Shafa Orthopedic Hospital, Iran University of Medical Sciences, Tehran, Iran; 2 Joint Reconstruction Research Center, Imam Khomeini Hospital Complex, Tehran University of Medical Sciences, Tehran, Iran

**Keywords:** Femoral head asphericity, Femoral head reduction osteotomy, Legg–Calvé–Perthes disease, Periacetabular osteotomy

## Abstract

**Background:**

The purpose of this study is to describe the midterm clinical and radiologic outcomes of concurrent femoral head reduction osteotomy (FHRO) and periacetabular osteotomy (PAO) in Legg–Calvé–Perthes disease (LCPD) patients with major aspherical femoral head deformities.

**Methods:**

The study included four Perthes patients in Stage IV of Waldenstrom’s classification with a mean age of 10.5 and severe femoral head asphericity. They were treated with a combination of FHRO + PAO and followed for at least 2 years. An evaluation of the radiological outcome of the surgery was carried out based on the lateral center to edge angle (LCEA), the anterior center to edge angle (ACEA), the Tönnis angle, the head sphericity index, the Stulberg classification, the extrusion index, and Shenton’s line integrity. An evaluation of the clinical outcome was made by evaluating hip range of motion (ROM), Harris hip score (HHS), and Merle d’Aubigne´-Postel score.

**Results:**

All radiographic measures improved; three patients were classified as Stulberg class II and one as class III. The LCEA, ACEA, and Tönnis angle improved by 29° (from 3° to 32°), 16° (from 14° to 30°), and − 10° (from 18° to 8°), respectively. The mean femoral head sphericity index and extrusion index improved by 12% (from 83 to 95%) and − 33% (from 40 to 7%). No disruption was observed in the postoperative Shenton’s line. According to HHS, all patients have shown excellent hip function, which improved by 27 points (from 69 to 96). Moreover, the hip ROM was increased from 222° to 267°. The follow-up period did not reveal any serious postoperative complications, such as osteonecrosis or conversion to arthroplasty.

**Conclusions:**

Combined FHRO with PAO may improve the hip joint’s morphology and function in patients with residual femoral head deformity and acetabular dysplasia due to LCPD. Despite being considered a complex and demanding hip surgery, these results suggest a more widespread implication of the salvage procedure.

## Introduction

In Legg–Calvé–Perthes disease (LCPD), the femoral head usually becomes aspherical and enlarged due to the acetabulum’s failure to contain it properly. LCPD could result in complex hip deformities, including coxa magna, coxa vara, femoral head central osteonecrosis, and abnormal greater trochanter anatomy [1, 2]. As a result of asymmetric enlarged femoral heads (coxa magna) not adequately contained by the acetabulum, femoroacetabular impingement (FAI) and hinged abduction occurred [3]. Eventually, altered hip biomechanics cause premature joint degeneration [4] and if hip preservation strategies do not work, these adolescents may have to undergo total hip resurfacing or arthroplasty [5–8].

A significant challenge for hip surgeons is the residual deformities associated with LCPD. There is still controversy regarding the treatment method for old-aged children with complex femoral deformities due to LCPD. In active stages of the disease (initial and fragmentation stages), femoral varus derotation osteotomy is an effective containment procedure since it prevents the femoral head from migrating laterally [9]. Aydin et al*.* [9] reported long-term [25-year follow-up] FVDO results on 21 hips of LCPD patients aged 6–12 years. A congruent joint was observed in 52% and no arthritis was found in 67% of hips; age lower than 10 and group A and B of the lateral pillar is associated with better clinical and radiological outcomes [9]. As proximal femoral osteotomies help contain the femoral head in the acetabulum, they are only partly effective in correcting intracapsular deformities, as the correction does not address the site of the deformity itself [10]. It is still possible for the femoral head to be incongruent with the acetabulum in this setting and to result in suboptimal results [10]. A new renaissance in the treatment of late Perthes disease was sparked by the development of the "safe surgical dislocation" method because it allowed the treatment of misshaped femoral heads with resection alone without causing avascular necrosis (AVN) [11]. In this respect, it has been recognized by Ganz et al*.* that the central third of a misshaped femoral head is the most damaged area. Accordingly, the central section of the femoral head was resected, and the two spherical lateral ends were brought together, while the vascular pedicle of the medial part was preserved. Through this technique, known as femoral head reduction osteotomy (FHRO), they were able to reshape the femoral head into a more spherical shape while also alleviating concerns about the development of AVN [12, 13]. This technique, however, cannot be used for asphericity in the frontal plane due to a potential risk of injury to the femoral head’s nutrient vessels [14].

In cases of severe acetabular dysplasia and FAI, a single FHRO may cause hip instability. Thus, acetabular reorientation with a periacetabular osteotomy (PAO) is being considered to prevent further instability [2, 14, 15]. It is necessary to correct both acetabular dysplasia-induced instability and FAI caused by femoral head deformities in complex cases [2]. A combined FHRO + PAO is designed to correct FAI and stabilize joints, improve patients’ symptoms, and preserve the native hip joint for the long term.

There is a lack of evidence on the outcomes of simultaneous FHRO and PAO surgery to preserve hip joints in patients with severe LCPD deformities. In this study, we review the midterm clinical and radiologic outcomes of combined FHRO + PAO procedures in a single operation in LCPD patients with major aspherical femoral head deformities.

## Methods

### Study design and setting

A Declaration of Helsinki was adhered to in the conduct of this study. Our institutional review board reviewed and approved this study. Written consent was obtained from the patient or the patient’s guardian before participation in the study. A retrospective review of prospectively collected hip surgery data between 2014 and 2020 was conducted to retrieve medical profiles of Perthes cases with major aspherical femoral head deformities (*n* = 31 consecutive cases). Those patients who underwent a combined FHRO and PAO procedure under one surgical anesthesia with a minimum of 2 years of follow-up were included in the study. This study was performed at the tertiary center of Shafa hospital, Tehran, Iran.

### Participants and criteria

According to Clohisy et al*.* [15], surgical indications of FHRO + PAO were the patients with age of < 20 years and symptomatic hip secondary to an aspherical and enlarged femoral head, central AVN of the femoral head, hinged abduction, and/or insufficient containment of the femoral head. Contraindications were severe incongruency that could not be corrected by FHRO, advanced cartilage disease, healthy central femoral head, and lateral and medial segments asphericity of the femoral head. This salvage procedure is only performed without other appropriate treatment options with predictable, satisfying outcomes.

### Surgical technique and postoperative rehabilitation

**Fig. 1:**
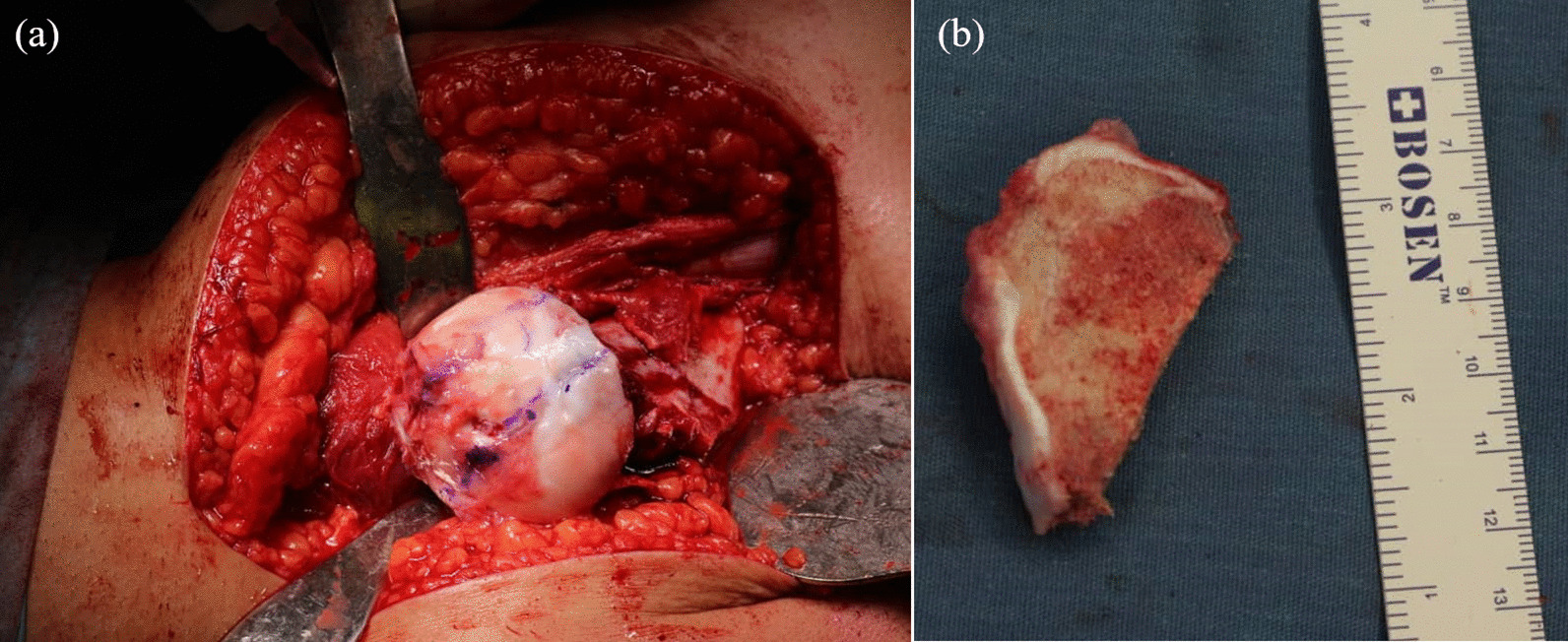
Intraoperative image **a** surgical dislocation and the central necrotic area, **b** osteotomy and resection of the central femoral head

The surgical technique was performed as previously described [11, 16]. Briefly, the patient was placed in a lateral decubitus position, and the hip was dislocated anteriorly using a flat trochanteric osteotomy In the gap between the gluteus maximus and medius (Gibson interval). There was a surgical dislocation of all hips, and an intraoperative dynamic assessment and radiologic examination were performed during the procedure [11, 12] (Fig. 1a). The FHRO performed first, followed by the PAO. Anatomical reshaping, lengthening of the femoral neck, and intra-articular lesions were addressed during the femoral head surgical dislocation. In this way, both intra-articular and extra-articular FAI could be addressed. For relative femoral neck lengthening (RFNL), the greater trochanter was trimmed down to the level of the superior femoral neck. Afterward, an extended retinacular soft tissue flap was formed, including the associated branches of the medial circumflex femoral artery, to ensure femoral head vascularity. The femoral head was then osteotomized in the sagittal direction to preserve the head’s vascularity (Fig. 1b). In the medial and lateral segments of the head adjacent to the necrotic region, articular cartilage and subchondral bone should still be retained, and the head should be shaped with relative sphericity when the reduction is made. A 6–12-mm-width necrotic section was removed from the center of the femoral head, followed by the lateral segment being reduced to the medial segment with care to avoid any articular step-off between the segments. The extended retinacular soft tissue flap and retinacular branches of the medial circumflex femoral artery also supplied blood to the mobile fragment. The osteotomy was then fixed with three 3.5 mm headless screws distal to the femoral head, and a bone graft was used to fill the gaps at the inferior margin of the lateral fragment. The mobile trochanteric segment was reduced and fixed after examining the dynamic hip range of motion (ROM) and removing all sources of inter- and extra-articular impingement.

**Fig. 2:**
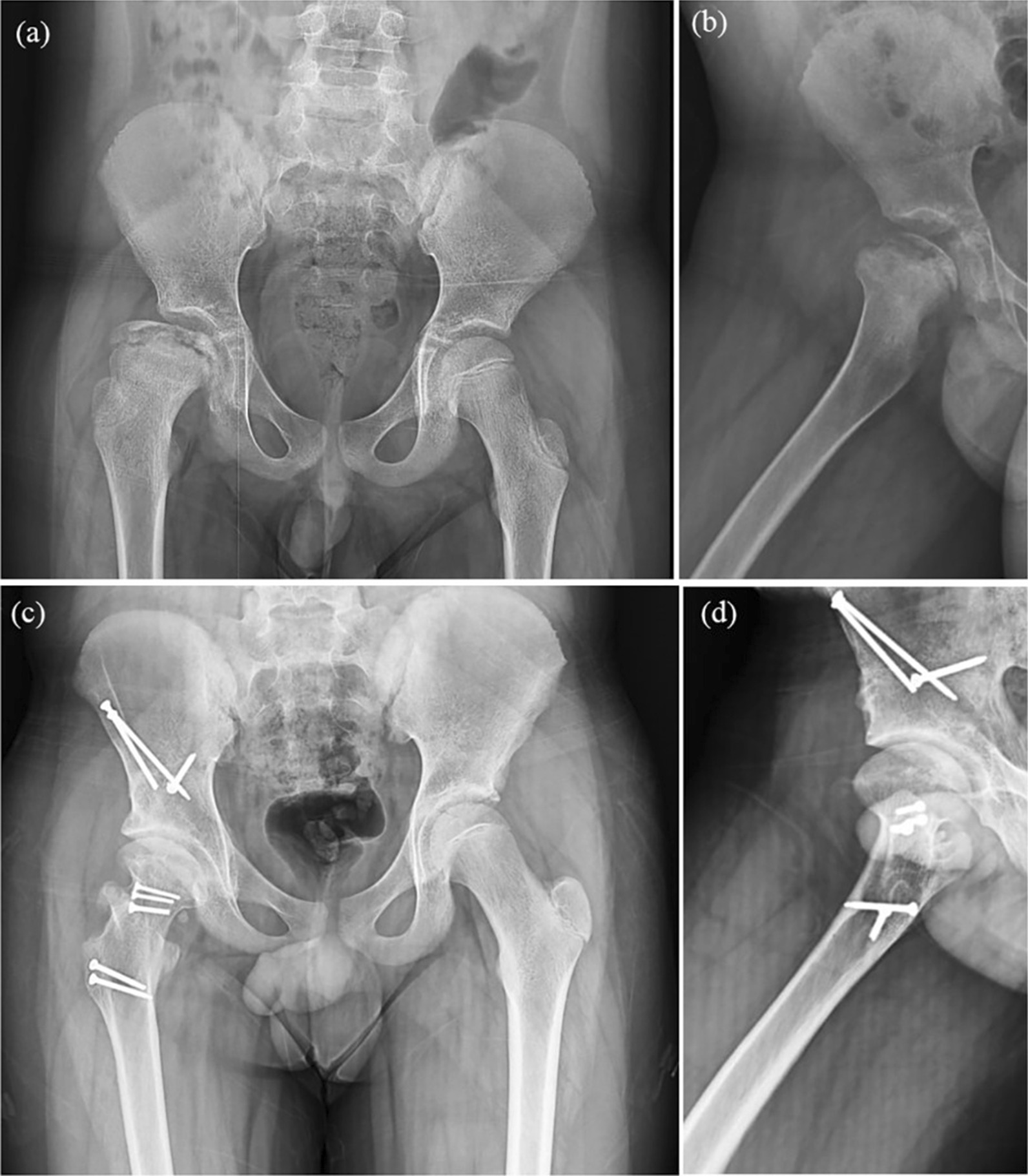
Case 1 (8 y/o boy) with FHRO + PAO (ITO), **a** Before surgery and **b** at the latest follow-up

**Fig. 3:**
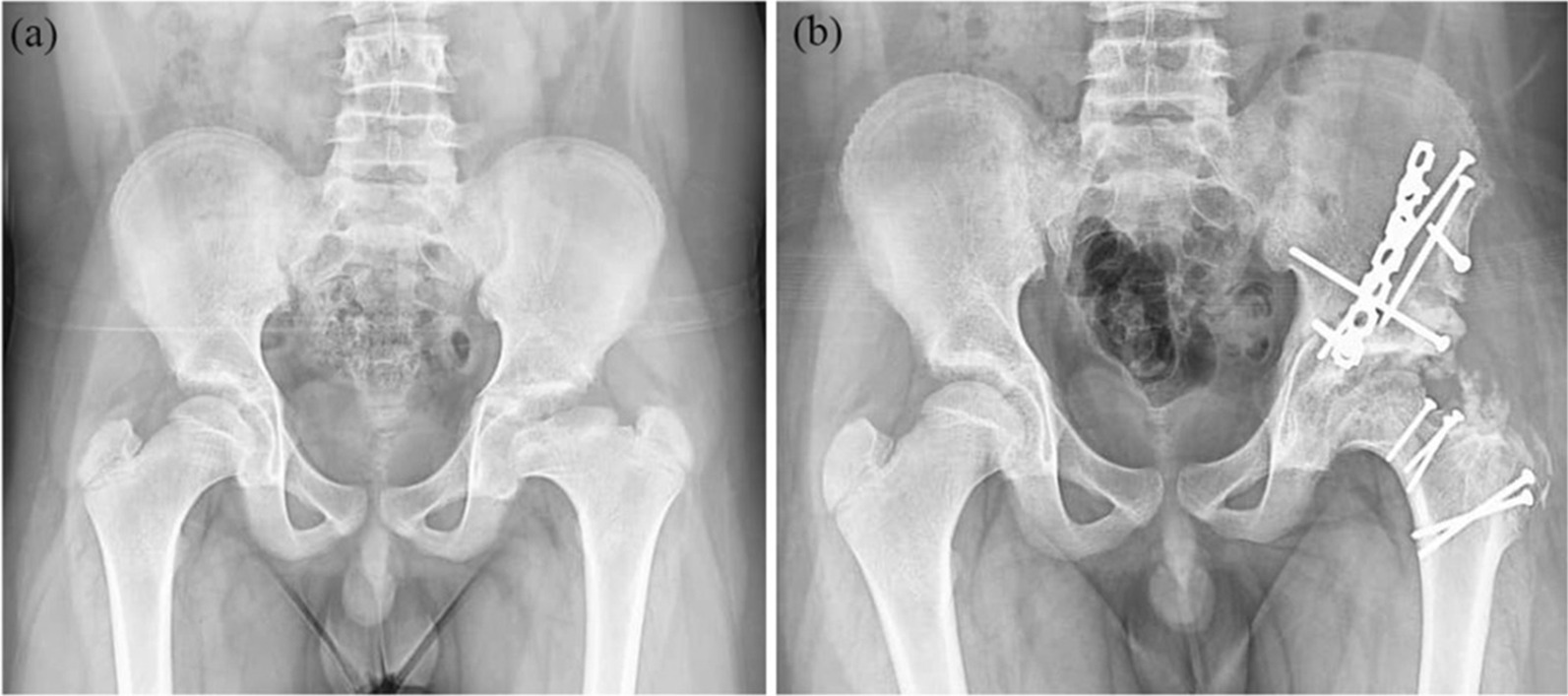
Case 4 (12 y/o boys) with FHRO + PAO (Ganz), **a **before surgery and **b** at the latest follow-up

Finally, concurrent PAO was performed using the triple innominate osteotomy or Ganz osteotomy technique as previously described [17, 18] (Figs. 2 and 3). It was determined that radiographic evidence of acetabular dysplasia and dynamic instability necessitated a PAO [1]. The deformity correction was checked intraoperatively using anteroposterior and false profile fluoroscopic images. Radiographic correction of the PAO was performed intraoperatively and ROM testing. Following the PAO, > 90° degrees of hip flexion was maintained to prevent secondary FAI.

After the operation, patients were restricted to partial weight-bearing for 2 months. During the first 4 weeks following surgery, patients were required to engage in continuous passive motion (CPM) and to limit hip flexion to 90°. If the hardware becomes symptomatic or interferes with the patient’s daily activities, it is generally recommended that it be removed after 6 to 12 months following surgery.

### Outcome measures and data collection

**Fig. 4:**
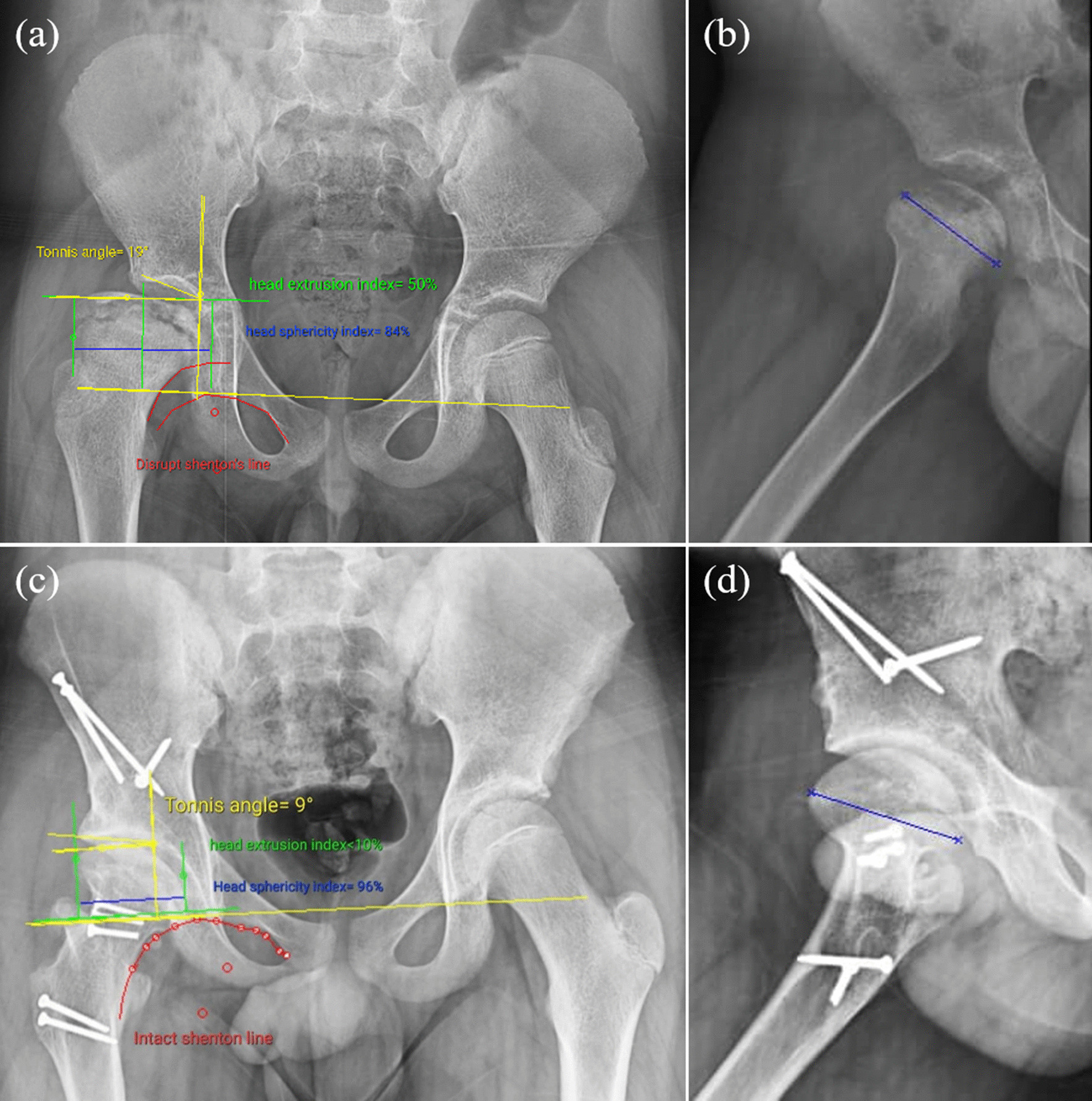
Improvement of radiological indexes by the combined FHRO + PAO procedures **a**, **b** before surgery and **c**, **d** at the latest follow-up (Tonnis angle: 19° to 9°; Extrusion index: 50% to 10%; Sphericity index: 84% to 96%; and Shenton’s line: disrupted to intact)

Before surgery, patients underwent radiological assessment, including X-ray radiography, MRIs, and CT scans with 3D reconstruction. The radiographs included an anterior–posterior (AP) pelvic radiograph, functional abduction, false profile hip, and a 45° Dunn view. The MRI reveals AVN in the central femoral head and osteochondral fragments and lesions. Furthermore, the CT scan helped with accurate head morphology and bone condition. To evaluate the radiographic characteristics of the patients, lateral center to edge angles (LCEA) were measured on AP radiographs postoperatively (aiming at 20° to 35° [19]) and anterior center to edge angles (ACEA) on false profile (aiming at 18° to 38° [20]). A Tönnis angle was used to measure the acetabulum’s weight-bearing surface [15]. The head sphericity index was used to assess femoral head sphericity [21]. Osteoarthritis degree was determined using the modified Tonnis classification, excluding the aspheric component since all hips have an aspherical head [22]. In addition, the final head morphology was investigated on the AP pelvic radiographs using the Stulberg classification and classified into five classes ranging from a normal hip (class I) to a flat femoral head with a normal acetabulum and neck (class V) [23–25]. The Shenton’s line and extrusion index were used to evaluate femoral head containment (Fig. 4).

Improvement in hip pain and function was assessed by Merle d’Aubigne´-Postel scoring [26], hip ROM using a goniometer (flexion, abduction, adduction, internal and external rotation in 90° of flexion), and Harris hip score (HHS) [27]. If the HHS was less than 70 points, it was considered symptomatic, but if it was greater than 80, it was considered good or excellent [28]. All the outcome measures were evaluated before the operation and at the last follow-up session by an expert hip and pelvis fellowship (** or **). According to the patient’s medical profiles, we have identified postoperative complications, including AVN of the femoral head, fracture of the femoral neck, non-union of the osteotomy sites, heterotopic ossification, and conversion to total hip arthroplasty.

Since there were a limited number of patients, no statistical analysis was performed to compare pre-and postoperative outcomes.

## Results

A total of four eligible patients (4 hips) were included in the study. Table 1 presents the characteristics of these patients. All patients were males, with a mean age of 10.5 years and a mean body mass index (BMI) of 24.3 kg/m^2^. Patients were followed up for an average of 5 years (range 2–8 years). According to Waldenstrom’s classification, all patients were in Stage IV (Late) and none were in active phase. All patients had an open proximal femoral physis. Two patients underwent PAO with triple osteotomies (Fig. 2), and two other patients underwent Ganz osteotomies (Fig. 3). The mean width of the resected bone was 10.8 ± 1.5 mm (Fig. 1b).

**Table 1:** Characteristic features of Perthes patients who underwent a combined femoral head osteotomy and periacetabular osteotomy (PAO)

Patient	Age at surgery	Sex	Laterality	BMI (kg/m^2^)	Follow-up (year)	PAO	Waldenstrom classification	Tonnis grade	Previous hip surgery	Resected length (mm)
1	8	Male	Right	23	8	Triple	Stage IV (Late)	1	Abductor tenotomy	10
2	10	Male	Right	26	6	Triple	Stage IV (Late)	1	None	10
3	12	Male	Right	23	2	Ganz	Stage IV (Late)	0	None	13
4	12	Male	Left	25	4	Ganz	Stage IV (Late)	1	None	10
Mean	10	–	–	24.3	5	–	–	–	–	10.8

*BMI* Body mass index, *PAO* Periacetabular Osteotomy

**Fig. 5:**
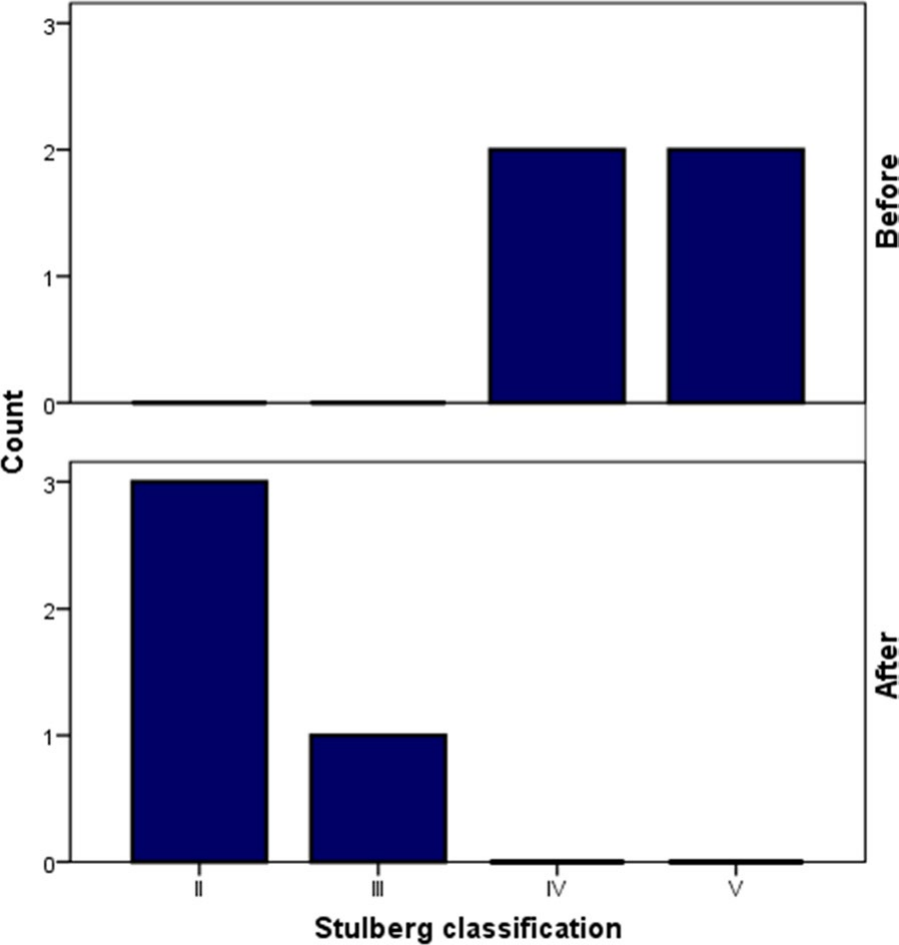
Improvement of Stulberg classification at the follow-up

The pre- and postoperative radiological outcome measures are demonstrated in Table 2. The LCEA and ACEA improved by 29.3° (from 2.8 ± 12.7° to 32.0 ± 2.5°) and 16.3 (13.8 ± 9.5° to 30.0 ± 3.3°), respectively. The Tönnis angle decreased by − 10° (from 18.0 ± 0.8° to 8.0 ± 2.1°) in the most recent follow-up. The mean femoral head sphericity index improved from 83.3 ± 1.7% to 95.0 ± 1.4% in the most recent follow-up (11.8% improvement) (Fig. 4). Regarding the morphology of the femoral head, two patients had aspherical congruent flat heads (Stulberg class IV), and two had aspherical incongruent flat heads (Stulberg class V). However, in the postoperative follow-up, three patients had Stulberg class II (spherical congruency), and one patient had Stulberg class III (aspherical congruency) (Fig. 5). Before the procedure, the extrusion index was 39.8 ± 11.8% and has improved to 7.0 ± 8.7% since the last follow-up. No disruption was observed in the postoperative Shenton’s line.

**Table 2:** Radiographic outcome measures of the patients before the operation and in the most recent postoperative follow-up

Variable	Patients 1		Patient 2		Patient 3		Patient 4		Mean ± SD		Improvement
Time point	Before	Follow-up	Before	Follow-up	Before	Follow-up	Before	Follow-up	Before	Follow-up	
LCEA (°)	− 7	30	17	33	10	30	− 9	35	2.8	32	29.3
ACEA (°)	0	34	15	26	20	30	20	30	13.8	30	16.3
Tönnis angle (°)	19	9	18	10	18	5	17	8	18	8	− 10.0
Sphericity index (%)	84	96	81	93	83	96	85	95	83.3	95	11.8
Stulberg classification	IV	II	IV	II	V	III	V	II	–	–	–
Extrusion index (%)	50	10	29	18	30	0	50	0	39.8	7	− 32.8
Shenton’s line	Disrupted	Non-disrupted	Intact	Non-disrupted	Disrupted	Non-disrupted	Intact	Non-disrupted	–	–	–

*LCEA* Lateral center to edge angle, *ACEA* Anterior center to edge angle

**Fig. 6:**
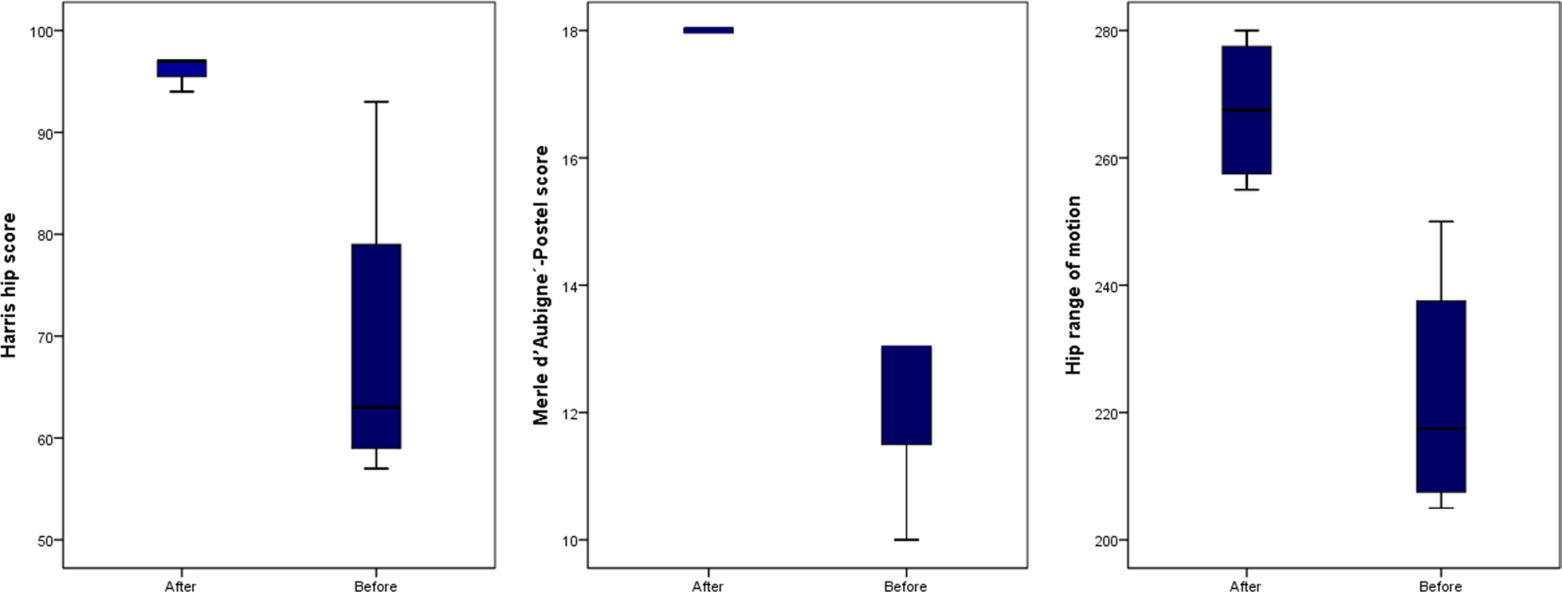
Improvement of functional scores (Harris hip score, Merle d’Aubigne´-Postel score, and hip range of motion

The pre- and postoperative clinical outcome measures are demonstrated in Table 3 and Fig. 6. The mean HHS improved by 27.3 points (from 69.0 ± 16.3 to 96.3 ± 1.5). The mean Merle d’Aubigne´-Postel score was 12.3 ± 1.5 before the operation and full (18 of 18) for all patients in the last follow-up. It was found that the hip ROM, defined as the sum of flexion, abduction, adduction, and internal and external rotation, was improved by 45° from 222.5° to 267.5°.

**Table 3:** Clinical outcome measures of the patients before the operation and in the most recent postoperative follow-up

Variable	Patients 1		Patient 2		Patient 3		Patient 4		Mean ± SD		Improvement
Time point	Before	Follow-up	Before	Follow-up	Before	Follow-up	Before	Follow-up	Before	Follow-up	
Harris hip score (HHS)	93	97	57	94	61	97	65	97	69	96.3	27.3
*Hip range of motion*											
Total	205	260	210	255	225	280	250	275	222.5	267.5	45
Flexion	120	120	120	120	130	130	120	120	122.5	122.5	0
Abduction	20	35	25	30	20	45	40	45	26.3	38.8	12.5
Adduction	10	30	10	30	20	30	30	30	17.5	30	12.5
Internal rotation	10	30	10	30	15	30	20	35	13.8	31.3	17.5
External rotation	45	45	45	45	40	45	40	45	42.5	45	2.5
*Merle d’Aubigne´-Postel score*											
Total	13	18	10	18	13	18	13	18	12.3	18	5.8
Pain	3	6	2	6	3	6	3	6	2.8	6	3.3
Mobility	6	6	5	6	6	6	6	6	5.8	6	0.3
Walking ability	4	6	3	6	4	6	4	6	3.8	6	2.3

No cases of hip AVN, femoral neck fracture, and infection were recorded in our patients. In all patients, heterotopic ossification was observed. Three showed a bone spur at the upper border of the femoral neck (Brooker class II), and the fourth patient had a mild case (Brooker class I). All patients had a complete union of the osteotomy site within 12 weeks at the greater trochanter, the acetabulum, and the femoral head and neck. None of the patients required early conversion to THA or further surgeries during the follow-up period.

## Discussion

In this study, we evaluated the radiologic and clinical outcomes of four consecutive FHRO + PAO as salvage procedures in patients with severe residual hip deformities due to LCPD. The main findings are that there was a substantial improvement in all of the radiographic measures evaluated in the most recent follow-up compared to the preoperative study, including the LCEA, ACEA, Tönnis angle, sphericity index, Stulberg classification, extrusion index, and Shenton’s line integrity. Furthermore, clinical outcomes, including hip ROM, HHS, and Merle d’Aubigne´-Postel score, have improved remarkably, and all patients have demonstrated excellent hip function according to HHS. No serious postoperative complications or the need for a second surgery were observed during the follow-up period.

The deformed femoral head has been an unresolved issue in orthopedics, leading to pain, limps, limitations in mobility, impingement, dysplasia of the acetabulum, and arthritis. In some cases, valgus and valgus-extension osteotomies can resolve symptoms and may lead to the preservation of the hip for a long time [29]. In recent years, the development of safer and more extensive cheilectomy techniques has opened up new possibilities for treating femoroacetabular impingement [12, 13, 16, 30]. In recent years, the Ganz safe surgical dislocation method has been combined with an FHRO based on the vascular anatomy of the femoral head, allowing non-spherical femoral heads to regain their sphericity [12, 13, 16, 30]. However, these patients are vulnerable to high instability as a high proportion has dysplastic acetabulum and insufficient containment [14, 16]. To minimize this risk and prevent further containment surgeries, we performed a concurrent FHRO + PAO to correct FAI and stabilize the joint previously reported in a few studies [14, 15]. The outcome of simultaneous FHRO + PAO surgery in patients with severe LCPD deformities is not well documented, and only three studies [16 hips] have addressed this matter (Table 4).

**Table 4:** Characteristics and outcomes of studies on combined FHRO + PAO in the literature

Study and reference	Year	Hips	Sex (boy)	Mean age (years)	Follow-up (year)	Open physis (%)	PAO	Previous hip surgery	Radiologic outcomes (before to after surgery)	Stulberg classification	Function	Hip ROM	Complications
Ganz et al	2011	14 (8 FHRO + PAO)	NR	NR	> 3 years	NR	PAO (*n* = 8)	NR	NR	NR	The motion was improved without significant pain for all patients	NR	Subsequent surgical procedures (*n* = 4): varus intertrochanteric osteotomy (*n* = 1) and PAOs (*n* = 3)
Siebenrock et al	2015	11 (4 had PAO + FHRO)	7 (64%)	13	5	1 (9.1%)	Triple (*n* = 2) PAO (*n* = 2)	Varus intertrochanteric osteotomy (*n* = 1)	Head sphericity:72 to 85% Extrusion index: 47 to 20% LCEA: 1 to 26° Shenton’s line: All intact (100%) Acetabular index: 17 to 7° Centrum–collum–diaphyseal angle: 133 to 139° Axial alpha angle: 40 to 42°	Before: V (*n* = 2) IV (*n* = 2) After: II (*n* = 5) III (*n* = 4) IV (*n* = 1)	Merle d’Aubigne´-Postel score: 14.5 to 15.7	Flexion: 94 to 91 Internal rotation: 13 to 15 External rotation: 28 to 23	Subsequent surgical procedures (*n* = 7): hardware removal (*n* = 2) and containment surgery (*n* = 5) Heterotopic ossification Brooker Grade II (*n* = 1)
Clohisy et al	2018	6 (5 with LCPD)	0	13.6	3.3	NR	*n* = 4	NR	LCEA: 2.5° to 30.6° ACEA: 18. 2° to 35.2° Tönnis: 20.1° to 2.5° Extrusion index: 45.3 to 13.6% α angle (Dunn view): 68.5 to 40.2° α angle (frog lateral view): 65.7 to 32.7°	Before: IV (*n* = 6)	HHS: 53.5 to 83.4 WOMAC: 62.3 to 90.3	NR	wound infection (*n* = 1)
Present study	2022	4	4 (100%)	10	5	4 (100%)	Triple (*n* = 2) Ganz (*n* = 2)	Abductor tenotomy (*n* = 1)	LCEA: 2.8° to 32° ACEA: 13.8 to 30° Tönnis: 18° to 8° Sphericity index: 83.3 to 95% Extrusion index: 39.8 to 7% Shenton’s line: All non-disrupted	Before: V (*n* = 2) IV (*n* = 2) After: II (*n* = 3) III (*n* = 1)	HHS: 69 to 96.3 Merle d’Aubigne´-Postel score: 12.3 to 18	Total: 222.5° to 267.5° Flexion: 122.5 to 122.5 Abduction: 26.3 to 38.8 Adduction: 17.5 to 30 Internal rotation: 13.8 to 31.3 External rotation: 42.5 to 45	Heterotopic ossification (*n* = 3 grade II and *n* = 1 grade I)

*FHRO* Femoral head reduction osteotomy, *PAO* Periacetabular Osteotomy, *HHS* Harris hip score, *NR* not reported

Clohisy et al*.* [15] reported the outcomes of a combined FHRO + PAO for treating six patients with severe femoral head deformities with a mean follow-up of 3.3 years (Table 4). Radiographic measures, including LCEA, Tönnis angle, medial offset, extrusion index, and α angle, were significantly improved in the last follow-up. A significant improvement was also observed in the HHS and WOMAC scores in the last evaluation. There was no need to convert to THA or to perform additional surgery for any of the six hips, and only one wound infection was treated with irrigation and debridement. However, two patients had poor or fair function regarding HHS, and one reported experiencing pain after participating in sports activities. As a result, they concluded that FHRO combined with PAO results in significant improvements in clinical and radiological outcomes in short-term follow-up [15]. Although their results were similar to ours, we did not observe a poor functional outcome among the patients. Moreover, Stulberg class was not reported to detect the sphericity and congruency of the femoral head since this classification could be used to predict the long-term outcome [31]. Three class II and one class I Stulberg at the latest follow-up could predict good long-term results.

Another study by Siebenrock et al*.* [14] reports FHRO with concomitant RFNL, five of which had simultaneous containment surgery, including two PAOs, two ITOs, and one Colonna procedure at the index surgery. However, five other patients required further surgeries to improve containment, including one PAO and three ITOs. They noted that FHRO could improve femoral head sphericity, but acetabular containment surgery is also required in these hips with dysplastic acetabulum, ideally concurrently [14]. There were seven cases without concurrent containment surgery, of which five required it after a mean of 2.3 years (three ITOs, one intertrochanteric varus osteotomy, and one PAO). Once again, neither AVN nor conversion THA was reported.

Ganz reported additional containment procedures were performed in 13 of 14 hips. Of these, nine were concurrently performed together (one Colonna procedure and eight PAOs), and four were performed later (one varus intertrochanteric osteotomy and three PAOs) [13] (Table 4). It is expected that additional containment surgery will be necessary at the time of FHRO rather than a subsequent procedure. The presence of adequate femoral head containment in open physis cases, such as our study, allows the head to remodel optimally [14].

However, some authors have also reported that FHRO can be performed without the need for containment surgery such as PAO. The Ganz technique of FHRO was utilized by Paley et al*.* in 20 patients to reduce the size and reshape the femoral head, and three patients underwent pelvic osteotomies (Wagner 1 types) concurrently with the FHRO, and two patients underwent Ganz PAO 6 months after the index procedure [32]. Five patients used an external fixator to maintain the femoral head within the acetabulum fossa during the first 6 weeks following surgery. An improvement of sphericity from 133 to 96% was observed when the diameter of the femoral head of the surgical side was divided by the diameter of the healthy side. Of the 20 patients, 14 had good or excellent functional and radiographic results, while six had suboptimal outcomes, and one had an AVN [32]. The patient with AVN was the only one with an open physis. They recommended that a persistent open physis might therefore contraindicate this procedure. However, none of the patients in the present series experienced pain, stiffness, or limitations in hip ROM at their last follow-up. This difference could be attributed to the fact that our patients underwent combined PAO. We have demonstrated that FHRO can also be performed on hips with an open epiphysis without the development of AVN, and based on the previous studies rate of AVN is rare [14, 16, 29].

There were a number of limitations to the present study. As a result of the anatomical deformities present in these cases, combined FHRO + PAO is a demanding procedure with a significant learning curve. As a result, this technique should be promoted cautiously and only in appropriate cases. Despite including all eligible patients of our orthopedic referral center, the study was limited by the small number of patients. In this regard, all of the cases had open physis, and the outcomes of patients with closed physis could not be provided. Moreover, PAO was not performed in the same manner for all patients. A retrospective design could also be considered a limitation of the study, leading to bias in the results. Last but not least, the short follow-up of the patients (mean 5 years) is insufficient for concluding if there will be osteoarthritis and a need for hip replacement. Thus, future standard prospective studies with larger patient populations could provide a more accurate picture of concurrent FHRO + PAO outcomes for patients with complex residual deformities.

## Conclusion

The combination of FHRO and PAO procedures in a single operation has demonstrated remarkable improvement in the clinical and radiographic index of patients with complex residual deformities of LCPD. It could be viewed as a salvage procedure for enlarged misshapen femoral heads accompanied by central necrosis and hip dysplasia with minimal complications and AVN sequelae. However, it needs to be confirmed in future studies with long-term follow-ups and a larger number of patients.

## Data Availability

Represented in the study tables.
